# Effect of Corn Processing and Protein Degradability on Ruminal Metabolism and Feeding Behavior of Dairy Cows

**DOI:** 10.3390/ani16010107

**Published:** 2025-12-30

**Authors:** Danielle de Cássia Martins da Fonseca, Cristian Marlon de Magalhães Rodrigues Martins, Bruna Gomes Alves, Carlos Eduardo Fidelis, Marcos Veiga do Santos

**Affiliations:** Department of Animal Nutrition and Production, School of Veterinary Medicine and Animal Science, University of São Paulo, Pirassununga, São Paulo 13635-900, Brazil; dmartinsfonseca@usp.br (D.d.C.M.d.F.); cristian.martins@alumni.usp.br (C.M.d.M.R.M.); brunagomesufu@gmail.com (B.G.A.); carlosfidelis@usp.br (C.E.F.)

**Keywords:** *Bos taurus*, digestibility, ground corn, nitrogen, starch, steam-flaked corn

## Abstract

This study investigated how corn processing and protein degradability affect ruminal fermentation and feeding behavior in lactating Holstein cows. Twenty cows (averaging 162 ± 70 days in lactation, 666 ± 7 kg body weight, and 36.0 ± 7.8 kg/day milk yield) were assigned in a Latin square design with four 21-day periods and four treatments arranged in a 2 × 2 factorial: corn processing [ground corn (GC) vs. steam-flaked corn (SFC)] and crude protein (CP) degradability [high (HCP) vs. low (LCP)]. Ruminal samples were collected at eight time points (0, 2, 4, 6, 8, 10, 12 and 16 h) post-feeding to analyze pH, ammonia, and short-chain fatty acids, while feeding behavior was recorded visually every 5 min for 48 h. Corn processing and protein degradability interacted to influence rumen ammonia nitrogen (*p* = 0.057), urinary pH, (*p* = 0.041), nitrogen secretion and efficiency (*p* = 0.538), and feeding (min/kg DM; *p* = 0.049) and rumination times (min/kg DM, *p* = 0.001; min/kg NDF, *p* = 0.001), reflecting changes in nitrogen metabolism. Steam-flaked corn decreased the acetate/propionate ratio and enhanced propionate production, improving nitrogen retention and reducing urinary N losses, while highly degradable protein increased ruminal NH_3_-N and branched-chain VFA concentrations, particularly when combined with ground corn. Additionally, steam flaking reduced feed selectivity and increased rumination efficiency, supporting more effective use of nutrients for milk N secretion and overall nitrogen utilization efficiency in dairy cows. Overall, diets varying in corn processing and protein degradability altered ruminal metabolism, nutrient utilization, feeding behavior, and diet selectivity in lactating cows, highlighting their importance in optimizing dairy cow performance.

## Simple Summary

In Brazil, the main corn used for dairy cows feeding has a highly vitreous endosperm, which can reduce digestibility because starch granules are embedded in a dense protein matrix. Processing techniques such as grinding or flaking can improve ruminal starch availability and energy use. In this study, we evaluated how corn processing and protein degradability influence ruminal fermentation and feeding behavior in lactation Holstein cows. In general, steam-flaked corn increased concentrations of propionate and reduce acetate/propionate ratio in rumen. Our findings demonstrate that both corn processing and protein degradability affect nutrient utilization and feed selection, emphasizing the importance corn processing optimization and protein degradability to improve milk production efficiency and nitrogen use in dairy herds.

## 1. Introduction

Corn (*Zea mays* L.) is a globally cultivated crop with diverse applications. According to USDA data, the 2025/2026 harvest is projected to yield approximately 1.26 billion tons of corn worldwide, with Brazil contributing approximately 131.0 million tons [1]. Approximately 50% of Brazilian corn production serves as animal feed, representing one of the primary components in animal diets, especially for cattle, and providing an important energy source for ruminal microbial growth and development [1,2].

In the main regions of the world, most cultivated corn is of the floury type (*Dent Zea mays* ssp.). In Brazil, however, the cultivated corn is predominantly of the flint type (*Flint Zea mays* ssp.) The main difference between floury and flint corn relates to the endosperm. The starch in the endosperm of flint corn is almost entirely rigid (glassy), while floury corn has a large part of its starch as floury endosperm. Thus, corn hybrids differ in their ratio of glassy to floury endosperm [3].

Corn is the main energy source used in dairy cow diets, but its nutritional contribution depends directly on the physical and chemical characteristics of the kernels. In Brazil, hard-kernel (flint) corn predominates, a result of historical breeding efforts aimed at increasing resistance to environmental stresses and pests. This type of corn has greater endosperm vitreousness, a higher proportion of protein matrix surrounding the starch granules, and lower structural fragility factors that reduce its ruminal and intestinal digestibility compared with dent corn commonly used in temperate regions. Consequently, the effective availability of starch to cows is often limited, especially when grain processing is inadequate [4,5].

In tropical dairy production systems, this limitation is even more relevant. The greater dependence on corn as the primary energy ingredient, combined with climatic conditions that promote heat stress and reduce dry matter intake, makes starch-use efficiency a critical factor for sustaining milk production. Moreover, the variability in the quality of commercially available flint corn increases the heterogeneity of productive responses, requiring greater attention to the type and intensity of processing. Thus, understanding the nutritional limitations of Brazilian hard corn is essential for guiding processing strategies, diet formulation, and the optimization of feed efficiency in tropical environments [6].

The majority of corn produced in Brazil is the hard or “flint” type due to its superior adaptability to tropical climates and greater resistance to insect attack during storage [3]. However, this variety possesses a vitreous endosperm and dense protein matrix, which render it resistant to digestive enzymes, thereby hindering ruminal microorganism access to starch, the primary energy source [7].

Grain processing methods significantly impact nutritional value [8]. Traditional methods, such as grinding, and advanced techniques, such as steam flaking, alter the grain’s physical structure, thereby influencing nutrient intake, digestibility, feed efficiency, and animal performance [9,10].

Balanced carbohydrate and protein supply enhances animal performance and feed efficiency while reducing environmental impacts from dairy operation effluents. To satisfy microbial protein requirements without excess nitrogen, lactating cow diets must be balanced for rumen degradable protein (RDP) and rumen undegradable protein (RUP) requirements [11]. Although both corn processing and protein degradability are known to influence nutrient utilization in dairy cows, little is known about their combined effects particularly with flint corn prevalent in tropical regions. The physical morphology of corn, determined by endosperm structure, vitreousness, and the association between starch and proteins, directly influences the rate of protein and starch degradation in the rumen. Starch is organized in a matrix with zeins, which forms a hydrophobic “shield” and limits microbial access to both starch and protein. This barrier reduces ruminal digestibility, making grain processing such as grinding or fermentation necessary to break the starch protein matrix [12]. Processing increases surface area and exposes starch and zeins, simultaneously enhancing starch digestibility and protein degradation [13,14]. Additionally, supplementation with exogenous α-amylase can accelerate the breakdown of these complexes, further improving microbial access and ruminal digestion. This study addresses the question of different methods of corn processing (grinding versus steam flaking) and levels of protein degradability interact to influence ruminal fermentation, feeding behavior, and nitrogen utilization in lactating Holstein cows. The hypothesis of this study is that steam-flaked corn would increase starch degradability in the rumen, resulting in greater production of fermentable carbohydrates and improved ruminal fermentation profiles (e.g., lower acetate/propionate ratio and reduced ruminal NH_3_-N peaks). Furthermore, we expected that, when combined with a high RDP:RUP ratio (67.5% RDP and 32.5% RUP as a proportion of CP), nutrient utilization efficiency would be enhanced through improved synchrony between energy and nitrogen release, leading to more efficient microbial protein synthesis, reduced urinary nitrogen losses, and positive shifts in feeding behavior that support optimal rumen function in lactating dairy cows. Therefore, this study aimed to investigate the effects of corn processing and protein degradability on ruminal fermentation and feeding behavior in lactating Holstein cows. This study provides relevant insights for the combined influence of steam-flaked corn and dietary protein degradability on digestion, which has been insufficiently investigated under flint-type corn conditions.

## 2. Materials and Methods

All experimental procedures followed ethical principles for animal experimentation and standards established by the National Council for the Control of Animal Experimentation (CONCEA). The study was approved by the Animal Use Ethics Committee of the School of Veterinary Medicine and Animal Science, University of São Paulo (CEUA/FMVZ), and registered under CEUA No. 9571150916.

### 2.1. Experimental Design

Animal selection, treatment descriptions, ingredient proportions, and diet chemical composition were previously described by Martins [10]. At the beginning of the experiment, 20 Holstein cows (162 ± 70 DIM; 666 ± 7 kg BW; 36 ± 7.8 kg/d milk yield) were assigned to a balanced 5 × 4 Latin square design with four 21 d periods and four treatments in a 2 × 2 factorial arrangement. The cows were previously classified according to milk yield, days in milk, parity, and body condition score to form homogeneous groups of 4 animals within each square. After stratification, treatments were allocated by random draw in the first period to ensure unbiased assignment. In subsequent periods, treatments were rotated within each square according to the Latin square structure, ensuring that all cows received all treatments and minimizing potential biases related to animals or periods. Treatments consisted of 2 corn processing methods (ground corn, GC: 2 mm screen; 952 ± 1.86 μm particle size; or steam-flaked corn, SFC: 280 g/L flake density) and 2 CP degradability levels [high: 107 g RDP/kg DM and 51 g RUP/kg DM; low: 95 g RDP/kg DM and 63 g RUP/kg DM]. Each period included 14 d of adaptation and 7 d of sampling. Diets were based on corn silage (49.5% DM) and formulated as TMR to meet or exceed NRC (2001) requirements, except for low CP degradability, which resulted in negative ruminal protein balance (−75 g/d). Feed was offered ad libitum (5–10% refusals). Cows were housed in individual free-stall pens with sand bedding, fed twice daily (0800 and 1300 h), milked twice daily (0600 and 1600 h), and weighed on d 21 of each period.

### 2.2. Ruminal Metabolism

Ruminal fluid samples were manually collected using a flexible esophageal tube (approximately 100 mL per timepoint) to obtain a representative composite sample, specifically designed for cattle, attached to a vacuum-operated manual pump. Sampling was performed before (0 h) and at 2, 4, 6, 8, 10, 12, and 16 h after the morning feeding on the last day of each experimental period. To minimize saliva contamination, the first portion of the aspirated fluid (about 50 mL) was discarded, and samples were collected from the central ruminal area using a flexible oro-ruminal probe while avoiding the esophageal region. Ruminal pH was measured immediately after sampling using a portable pH meter (TEC-3P-MP, Tecnal^®^, Piracicaba, Brazil).

Ruminal pH was determined immediately after collection using a portable TEC-3P-MP pH meter (Tecnal^®^). Ruminal samples were submitted to centrifugation at 3000 RPM × 15 min. Subsequently, 1.6 mL of supernatant was transferred to Eppendorf tubes (Axygen^®^ Brand Products, New York, NY, USA) containing 0.4 mL of formic acid (P.A.) and stored at −20 °C for short-chain fatty acid (SCFA) and ammonia nitrogen (NH_3_-N) analyses. SCFA concentrations were determined in triplicate using gas chromatography (GC-2014, Shimadzu, Kyoto, Japan) using a Stabilwax^®^ capillary column (Restek, Bellefonte, PA, USA; 30 m × 0.53 mm) at 145 °C (isothermal) with a split/splitless injector and dual FID detection at 250 °C, following the method described by Erwin et al. [15]. Ruminal NH_3_-N concentration (mg/dL) was determined in triplicate using spectrophotometry according to Bergmeyer [16].

### 2.3. Fecal and Urinary Variables

Fecal and urinary sampling was conducted using spot sampling at 9h intervals over 3 consecutive days of each experimental period. Fecal samples (approximately 500 g/cow) were collected directly from the rectum, immediately after ruminal fluid sampling, weighed, diluted in 50 mL of distilled water. Ruminal and fecal pH were measured using a portable pH meter (TEC-3P-MP, Tecnal^®^, Brazil). Urine samples (approximately 200 mL/cow) were collected by vulva stimulation at time 0, 3, 6, 9, 12, 15, 18 and 21 h. A 20 mL subsample was diluted in 80 mL of 0.036 N H_2_SO_4_ to prevent NH_3_ volatilization and stored at −20 °C for later nitrogen analysis. Nitrogen balance and excretion were determined according to the methodology described by Harvatine and Allen [17], based on N intake, fecal and urinary N concentrations quantified using the Kjeldahl method.

### 2.4. Feeding Behavior

Feeding behavior was assessed using methodology adapted from Bürger [18] to evaluate treatment effects on cow performance. Behavioral observations were conducted on days 17, 18, and 19 of each experimental period. Cows were individually housed in free-stall systems and had ad libitum access to feed and water. Feeding behavior was recorded every 5 min for 48 h by five trained observers positioned to avoid interfering with animal activity. Observers were positioned in fixed locations designed to avoid disturbing the animals and to maintain consistent visibility across all observation periods. These standardized procedures were implemented to minimize observer bias and ensure reproducibility of behavioral measurements [19]. Variables analyzed included: (a) feeding time (FT, min); (b) rumination time (RT, min); (c) rumination per kg ingested DM (RT/kg ingested DM); (d) rumination per kg consumed NDF (RT/kg consumed NDF); (e) idle time (IT, min), representing time when animals were resting (lying or standing), interacting with other animals, or at feeding/drinking/housing structures; (f) water intake time (WIT); and (g) other activities (OAT), representing activities not fitting the above categories. Total chewing time (TCT, min) was calculated as the sum of FT and RT [20].

### 2.5. Particle Selection

To determine dietary particle size distribution, a Penn State Particle Size Separator (PSPS) was used, following the methodology of Lammers et al. [21]. Approximately 1000 g of diet samples were weighed, transferred to the Penn State particle separator, and manually shaken. Material was separated into four particle size categories based on sieve diameter: long (>19.0 mm), intermediate (<19.0 and >8.0 mm), short (8.0 to 1.18 mm), and very short (<1.18 mm) particles [21,22]. Diet selection index (DSI) was calculated using the methodology described by Leonardi and Armetano [20]:

$$\begin{array}{l} \mathrm{Predicted}\;\mathrm{intake}\;=\;\%\;\mathrm{retained}\;\mathrm{in}\;\mathrm{Px}\;\mathrm{of}\;\mathrm{the}\;\mathrm{offered}\;\mathrm{feed}\;\times \;\mathrm{total}\;\mathrm{intake}\\ \mathrm{Observed}\;\mathrm{intake}\;=\;(\%\;\mathrm{retained}\;\mathrm{in}\;\mathrm{Px}\;\times \;\mathrm{feed}\;\mathrm{offered})\;-\;(\%\;\mathrm{retained}\;\mathrm{in}\;\mathrm{Px}\;\times \mathrm{refusals})\\ \mathrm{Selection}\;\mathrm{Index}\;(\mathrm{SI})=\;(\mathrm{observed}\;\mathrm{intake}/\mathrm{predicted}\;\mathrm{intake})\;\times \;100 \end{array}$$

### 2.6. Statistical Analysis

Statistical analyses were performed using the MIXED procedure in SAS software (version 9.4, SAS Institute Inc., Cary, NC, USA). The MIXED procedure of SAS was used for data analysis according to the following model:

Yijklm = μ + Corni + CPdegj + (Corni × CPdegj) + Sk + Cl(k) + Pm + eijklm,

where Yijklm = dependent variable; μ = overall mean; Corni = fixed effect of corn processing i (1 df); CPdegj = fixed effect of CP degradability (1 df); Corni × CPdegj = fixed effect of interaction between Corni and CPdegj (1 df); Sk = fixed effect of Latin square k [1 to 5 (4 df)]; Cl(k) = random effect of cow l within each Latin square [l = 1 to 20 (15 df)]; Pm = fixed effect of period m [1 to 4 (3 df)]; and eijklm = random error associated with each observation.

The analytical model incorporated the fixed effects of corn processing method, protein degradability, their interaction, the Latin square design, and experimental period, as well as the random effect of individual cows within each Latin square. Model assumptions were verified by assessing the normality of residuals (via the Shapiro–Wilk test) and the homogeneity of variances (using Levene’s test). Degrees of freedom for fixed effects were determined using the Satterthwaite approximation to ensure accurate estimation in the presence of complex design factors. When significant effects or interactions were identified (*p* ≤ 0.05), Tukey’s HSD post hoc test was conducted for pairwise comparisons. Statistical significance was set at *p* ≤ 0.05 and statistical trends were considered for 0.05 < *p* ≤ 0.10.

## 3. Results

### 3.1. Ruminal Metabolism

**Figure 1:**
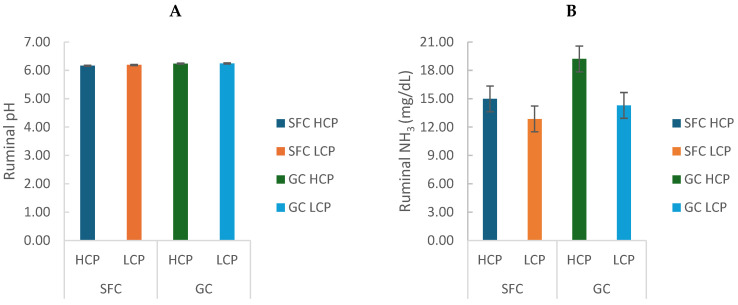
Effect of corn processing and crude protein degradability on ruminal pH (**A**) and N-NH_3_ concentration (mg/dL) (**B**).

Ruminal pH varied significantly throughout the day (6.21 ± 0.29; *p* < 0.0001) but was not affected by corn processing or protein degradability (Figure 1A). Ruminal ammonia concentration (N-NH_3_) was greater in cows fed ground corn (GC; 16.75 ± 3.47) compared with steam-flaked corn (SFC; 13.92 ± 1.50; *p* < 0.0001) and in cows fed high protein degradability diets (HCP) compared with low protein degradability diets (LCP; *p* < 0.0001). A trend for an interaction between corn processing and protein degradability was also observed (*p* = 0.058), with the highest N-NH_3_ concentrations in the GC-HCP treatment (Figure 1B).

**Figure 2:**
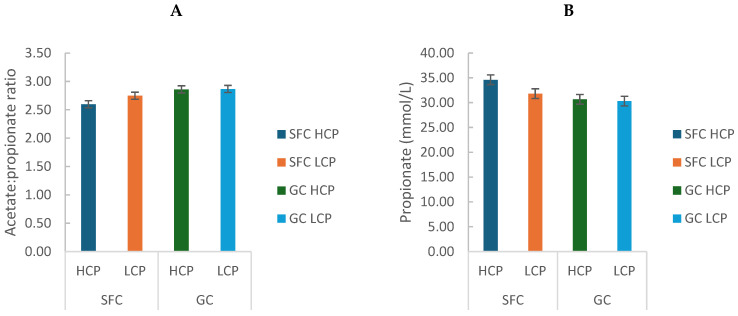
Effect of corn processing and crude protein degradability on acetate: propionate ratio (**A**) and propionate concentration (mmol/L) (**B**).

Corn processing reduced the acetate/propionate ratio (GC: 2.86 ± 0.01 vs. SFC: 2.67 ± 0.11; *p* = 0.0005) and increased propionate concentration (GC: 30.49 ± 0.25 vs. SFC: 33.21 ± 1.98; *p* = 0.002; Figure 2A,B). Total short-chain fatty acid (SCFA) concentration (µmol/L) varied only with sampling time (*p* < 0.0001; Figure S1), as did the concentrations of acetate (*p* < 0.0001; Figure S2A), butyrate (*p* < 0.0001; Figure S2B), and valerate (*p* < 0.0001; Figure S2C). Diets with HCP showed greater isobutyrate (*p* < 0.05; Figure S2D) and isovalerate concentrations (*p* < 0.05; Figure S2E).

Urinary pH was higher in cows fed LCP diets (*p* = 0.0046) and a significant interaction between corn processing and protein degradability was observed (*p* = 0.0413), with the lowest values in GC-HCP (Figure S3A). Fecal pH was higher in cows fed SFC (*p* = 0.001; Figure S3B).

### 3.2. Nitrogen Flow and Utilization

**Figure 3:**
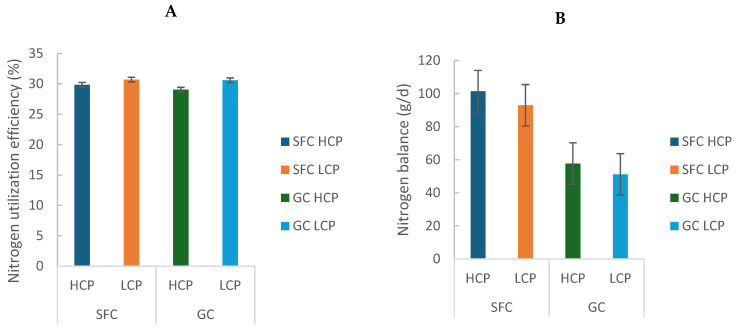
Effect of corn processing and crude protein degradability on nitrogen utilization efficiency (%) (**A**) and balance (g/d) (**B**).

Milk nitrogen secretion (g/d and % of N intake) was greater in cows fed LCP diets (GC: 172.72 ± 6.60 vs. SFC: 175.47 ± 3.59; *p* < 0.05). Urinary nitrogen excretion was greater in GC (141.00 ± 16.60) than SFC (122.24 ± 2.33; *p* = 0.009), and a trend for an interaction between factors was observed (*p* = 0.073), with the highest values in GC-HCP (Table 1). Fecal nitrogen excretion was also greater in GC (207.34 ± 8.32) than SFC (180.34 ± 2.33; *p* < 0.001). Nitrogen balance was more positive in SFC (97.21 ± 6.02) than GC (54.42 ± 4.61; *p* < 0.001; Table 1; Figure 3B), reflecting greater efficiency of nitrogen utilization for milk production (GC: 29.81 ± 1.09 vs. SFC: 30.27 ± 0.59; *p* = 0.002; Figure 3A).

### 3.3. Feeding Behavior

**Figure 4:**
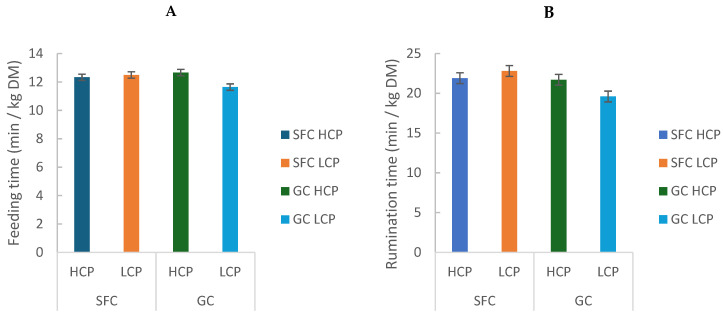
Effect of corn processing and crude protein degradability on feeding time (min/kg DM) (**A**) and rumination time (min/kg/DM) (**B**).

Daily feeding time was greater in cows fed GC (296.55 ± 1.34 min/d) than SFC (283.25 ± 0.07 min/d; *p* = 0.009; Table 2). Feeding time per kg of dry matter (DM) intake showed a significant interaction (*p* = 0.049), with the lowest values observed in GC-LCP (GC: 12.15 ± 0.72 min/kg DM; vs. SFC: 12.41 ± 0.11 min/kg DM; *p* = 0.009; Table 2; Figure 4A). However, despite statistical significance, the magnitude of the difference was small (approximately 12 min/d), which may indicate limited biological relevance.

Rumination time in min/kg DM (GC: 20.65 ± 1.48; vs. SFC: 22.35 ± 0.64) and rumination time in min/kg of NDF (GC: 2.72 ± 0.0; vs. SFC: 2,75 ± 0.01) was also affected by the interaction between factors (*p* = 0.0013 and *p* = 0.0019, respectively), with SFC-fed cows (HCP and LCP) having greater values than GC-LCP (Table 2; Figure 4B).

Water intake time was higher in LCP diets (*p* = 0.054), whereas other behaviors (idle time, lying time, standing time) were not affected (*p* > 0.10; Table 2).

### 3.4. Particle Selection

**Figure 5:**
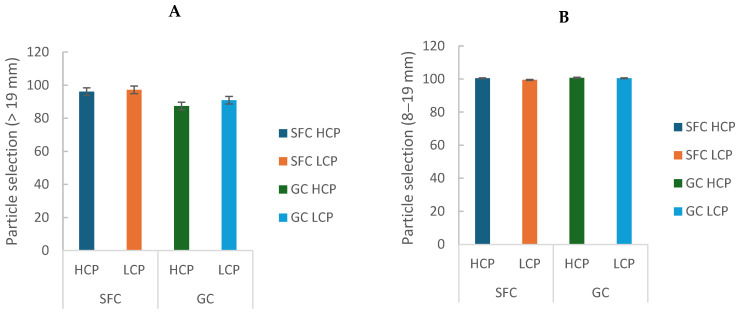
Effect of corn processing and crude protein degradability on the particle selection index for long particles (>19 mm) (**A**) and intermediate particles (8–19 mm) (**B**).

Refusal of long particles (>19 mm) was lower in cows fed SFC (*p* = 0.0009; Table 3; Figure 5A), indicating lower feed selectivity. There was also a trend for a lower preference for intermediate particles (8–19 mm) in cows fed SFC (*p* = 0.096; Table 3; Figure 5B). Other particle fractions were not affected (*p* > 0.10; Table 3).

## 4. Discussion

This study investigated the association between corn processing methods and protein degradability and their effects on ruminal metabolism, feeding behavior, and nitrogen utilization in lactating dairy cows, particularly relevant for systems utilizing flint corn prevalent in tropical regions.

### 4.1. Ruminal Metabolism

Our observation of marked temporal variations in ruminal pH, ammoniacal nitrogen (NH_3_-N), acetate/propionate ratio, and individual short-chain fatty acids (SCFAs), closely aligned with feeding times, reinforces the concept that rumen fermentation follows distinct diurnal kinetics driven by nutrient availability, microbial metabolism, and turnover [23], which can modulate rumen function and productive efficiency in dairy cows [24]. Moreover, the temporal nature of these processes may also alter daily patterns of methane emissions [25] and ruminal pH stability [26]. These consistent daily oscillations underscore the critical need for frequent sampling to accurately characterize rumen physiology and highlight the influence of feeding management on microbial activity and nutrient release in the rumen [27,28]. SFC likely increased propionic acid because starch from flaked corn undergoes greater ruminal gelatinization and has higher ruminal degradability compared with ground corn. This increases the availability of fermentable carbohydrates for amylolytic bacteria, which preferentially produce propionate via the succinate and acrylate pathways. Consequently, the more rapid and extensive fermentation of starch enhances the molar proportion of propionate in the rumen [29,30].

LCP increases milk nitrogen output because diets with higher levels of crude protein supply more rumen-degradable and rumen-undegradable protein, enhancing the amount of metabolizable protein available for absorption in the small intestine. Greater availability of amino acids improves mammary protein synthesis, thereby increasing milk true protein and total nitrogen secretion. When rumen nitrogen synchrony is adequate, this response reflects improved efficiency of dietary nitrogen use rather than increased nitrogen losses [31,32,33].

Our findings that corn processing and protein degradability did not significantly alter mean daily ruminal pH or total SCFA concentration align with previous research indicating that these factors may not always induce shifts in overall rumen acidity or fermentation intensity, especially when diets are well-buffered or physically effective fiber is adequate [34]. Results similar to those of the present study were found by Savari et al. [11], and Guyton et al. [35] observing that the type of corn processing and protein degradability had no effect on ruminal pH or VFA concentration.

However, ground corn (GC)-fed cows exhibited higher rumen N-NH_3_ concentration and acetate/propionate ratios compared to steam-flaked corn (SFC)-fed cows, while fecal pH was higher in SFC-fed cows. Similar results were obtained by Makizadeh et al. [36], who observed that the effect of the type of corn processing on N-NH_3_, VFA, and propionate concentrations was higher with SFC compared to GC, and that acetate concentration and acetate/propionate ratio were higher with GC compared to SFC. The elevated N-NH_3_ observed with GC likely reflects reduced synchronization between ruminal carbohydrate and nitrogen availability. Although finer grinding increases starch surface area and accelerates fermentation, the associated increase in acetate production indicates a more fibrolytic-driven fermentation pattern, which may not provide sufficient energy to support timely microbial incorporation of released ammonia. As a result, excess NH_3_-N accumulates in the rumen rather than being efficiently captured into microbial protein, reducing nitrogen-use efficiency and increasing losses through urine. In contrast, the greater propionate production and higher fecal pH observed with SFC suggest improved rumen fermentation balance and post-ruminal starch digestion, supporting more efficient microbial growth and nutrient utilization [37].

Conversely, SFC has often been associated with increased propionate production, reflecting enhanced starch gelatinization and increased ruminal bypass starch availability, ultimately improving energy efficiency [38]. Indeed, prior studies, including those by Zhong et al. [39] and Cooke et al. [40], have also shown that SFC can increase propionate concentration while concurrently reducing N-NH_3_ concentration and acetate/propionate ratio in ruminal fluid. This enhanced propionate production in SFC-fed cows may be associated with satiety mechanisms, as per the hepatic oxidation theory, through increased hepatic oxidation of energy compounds [34]. The link between increased ruminal propionate concentration and reduced dry matter intake, alongside the impact of cereal processing on starch digestibility and subsequent propionate production has been already an investigated aspect of ruminant nutrition [41].

During grinding, corn is reduced to smaller particles, increasing surface area and facilitating microbial adhesion and colonization, thereby improving starch degradation and ruminal fermentation efficiency. Ground corn often promotes faster fermentation, leading to increased ammonia production and resulting in higher, more variable N-NH_3_ concentrations. Additionally, finely ground corn can improve palatability and consumption ease, potentially influencing feeding time, while also promoting greater selection and rejection of long particles, contributing to digestion efficiency. In steam-flaked corn, lower acetate/propionate ratios are associated with greater efficiency in converting energy to lactose. In the present study, SFC-fed cows achieved the highest lactose production. Full details on milk production and composition results from this study are reported elsewhere [10].

N-NH_3_ concentration was higher in high crude protein degradability (HCP)-fed cows compared to low crude protein degradability (LCP)-fed cows. Additionally, individual SCFA concentrations and urinary pH were higher in LCP-fed cows compared to HCP-fed cows. Urinary pH was also higher in cows fed steam-flaked corn combined with both protein degradability levels compared to ground corn combinations. These observed effects of protein degradability on N-NH_3_ and SCFA concentrations are consistent with studies demonstrating that higher degradability leads to more rapid ruminal protein breakdown [42]. Highly degradable ruminal protein decomposes rapidly, reducing nitrate to nitrite and subsequently forming ammonia, thereby increasing ruminal N-NH_3_ concentration for microbial protein synthesis. This principle underlies grain processing strategies in animal nutrition, aiming to reduce ruminal protein degradability and ammonia losses to optimize nitrogen utilization efficiency [43].

Increased protein degradation alters branched-chain fatty acid profiles, which are microbial metabolism products contributing to energy production and ruminal acid-base balance. Moreover, increased ruminal protein degradability leads to elevated concentrations of branched-chain volatile fatty acids (BCVFAs), such as isobutyrate and isovalerate, which originate from the deamination and decarboxylation of branched-chain amino acids (valine, leucine and isoleucine) in the rumen microbial community [44]. These BCVFA are not merely by-products, but actively participate in microbial lipid synthesis and ruminal acid-base buffering, indicating that the profile of SCFA reflects both protein turnover and microbial metabolic integration of nitrogen and energy flows.

To maintain water balance, animals fed highly degradable protein diets tend to increase water consumption due to greater nitrogenous waste production. These digestive and metabolic changes in acid-base production can also alter urine pH. However, it is worth noting that some studies have observed no significant effects of protein degradability on ruminal pH, N-NH_3_, or VFA concentrations, highlighting the variable nature of responses depending on specific dietary compositions, basal diet characteristics, and experimental conditions [31]. Generally, increased protein supply or degradability is associated with elevated ruminal N-NH_3_ concentration [45].

### 4.2. Feeding Behavior

Feeding time (min/day) and intermediate particle selection index were higher in ground corn (GC)-fed cows compared to steam-flaked corn (SFC)-fed cows. This behavioral response may be related to the texture and palatability of the feed: diets with GC generally have a more fibrous and less cohesive structure, encouraging longer feeding and chewing times as cows select their preferred particles. In contrast, the softer, more gelatinous texture of SFC improved palatability and reduced chewing time due to lower requirements, resulting in faster meal consumption and less internal particle selection. These behavioral adaptations reflect how the holiday effort improves rumen function and feed comfort, balancing energy-rich components with physically healthy fibers [46].

Conversely, the long particle selection index was higher in SFC-fed cows compared to GC-fed cows. Water intake time was higher in low crude protein degradability (LCP)-fed cows compared to high crude protein degradability (HCP)-fed cows. Feeding time per kg dry matter, rumination time per kg dry matter, and rumination time per kg neutral detergent fiber were higher in SFC combinations (both HCP and LCP) compared to GC combinations. These findings on the effects of corn processing on feeding, rumination, and chewing times, as well as the observation that rumination and chewing times were shorter in HCP-fed cows compared to LCP-fed cows, are consistent with our previous work [47].

While some research indicates that an adequate provision of physically effective fiber might eliminate the need for additional forage-derived effective fiber, leading to minimal protein degradability effects on total chewing time or rumination activity, the present study’s results suggest that, even with adequate fiber, protein degradability can still influence behavioral patterns, potentially due to differences in metabolic load or satiety signals [48]. Increased rumination time per kg dry matter indicates the need for particle size reduction or enhanced saliva secretion to maintain rumen health and prevent acidosis, as greater rumination produces more saliva, thereby increasing ruminal buffering capacity [49]. The larger particle size of SFC compared to GC likely increases ruminal retention time, leading to increased rumination and chewing per kg dry matter [11].

The combined impact of corn processing method (ground vs. flaked) and ruminal protein degradability on feeding and rumination behavior is significant. Ground corn, being more finely processed, increases surface area and facilitates digestion, typically reducing feeding time due to easier consumption. Additionally, finely processed feeds generally transit the rumen more rapidly, potentially reducing rumination requirements. Conversely, highly degradable proteins decompose rapidly in the rumen, generating increased gas and waste production, which can influence rumination time as animals regulate pH and process metabolic byproducts. Low-degradability proteins are more resistant to microbial breakdown in the rumen, which may lessen the need for prolonged rumination. A slight increase in nitrogen intake (kg/day) was observed in SFC-fed cows compared to GC-fed cows (49.96% vs. 50.04%). The proportion of nitrogen secreted in milk (g/day) was also greater in SFC (50.39% vs. 49.61% in GC). Conversely, GC-fed cows showed higher urinary nitrogen excretion (g/day) (53.71% vs. 46.29% in SFC) and higher fecal nitrogen excretion (g/day) (53.48% vs. 46.52% in SFC). Rapid fermentation from ground corn combined with highly degradable protein not only decreases rumination time but may also reduce urinary pH due to increased metabolic acid excretion.

## 5. Conclusions

Corn processing and protein degradability markedly affected nitrogen metabolism in lactating dairy cows. Steam-flaked corn improved nitrogen utilization by reducing urinary nitrogen losses and increasing nitrogen retention, while ground corn elevated ruminal ammonia and nitrogen excretion. High protein degradability further intensified ammonia accumulation, particularly when combined with ground corn. Although behavioral changes were modest, the shifts in nitrogen metabolism were substantial. Practically, feeding steam-flaked corn with moderately degradable protein sources supports more efficient nitrogen use and may reduce environmental nitrogen excretion in dairy herds.

## Figures and Tables

**Table 1:** Effect of corn processing (SFC vs. GC) and protein degradability (HCP vs. LCP) on nitrogen flow and utilization.

Item	SFC-HCP	SFC-LCP	GC-HCP	GC-LCP	SEM	*p*-Value CP ^1^	*p*-Value PD ^2^	*p*-Value CP × PD ^3^
N intake (kg/d)	575.8	580.8	577.2	581.4	3.05	0.709	0.100	0.884
Milk N (g/d)	172.9	178.0	168.0	177.4	6.28	0.324	**0.013**	0.442
Urinary N (g/d)	120.6 ^b^	123.9 ^b^	153.6 ^a^	130.1 ^b^	8.20	**0.009**	0.169	0.073
Fecal N (g/d)	178.7	182.0	201.5	213.2	10.7	**<0.001**	0.299	0.557
Nitrogen balance (g/d)	101.5	92.9	57.7	51.2	17.5	**<0.001**	0.538	0.934
Milk N (% of N intake)	29.8	30.7	29.0	30.6	1.05	0.358	**0.022**	0.483
Urinary N (% of N intake)	20.9 ᵇ	21.4 ᵇ	26.6 ᵃ	22.2 ᵇ	1.32	**0.012**	0.255	0.267
Fecal N (% of N intake)	31.0	31.2	34.9	36.6	1.82	**<0.001**	0.423	0.565
N utilization efficiency (%)	28.5	29.3	27.0	27.1	0.88	**0.002**	0.449	0.538

^1^ CP = corn processing (SFC vs. GC). ^2^ PD = protein degradability (HCP vs. LCP). ^3^ CP × PD = interaction between the factors. Values followed by different letters differ (*p* < 0.05) according to the F test. The use of bold indicates the variables that show a statistically significant level (*p*-value < 0.05).

**Table 2:** Effect of corn processing and protein degradability on feeding behavior.

Item	SFC-HCP	SFC-LCP	GC-HCP	GC-LCP	SEM	*p*-Value CP ^1^	*p*-Value PD ^2^	*p*-Value CP × PD ^3^
Feeding time (min/d)	283.2	283.4	295.6	297.5	7.20	**0.009**	0.845	0.851
Feeding time (min/kg DM)	12.3 ᵃ	12.5 ᵃ	12.7 ᵃ	11.6 ᵇ	0.30	0.400	0.149	**0.049**
Rumination time (min/kg DM)	21.9 ᵃ	22.8 ᵃ	21.7 ᵃ	19.6 ᵇ	0.53	**0.0004**	0.158	**0.0013**
Rumination time (min/kg NDF)	2.76 ᵃ	2.74 ᵃᵇ	2.72 ᵇ	2.72 ᵇ	1.53	**0.004**	0.570	**0.0019**
Water intake time (min/d)	26.2	26.9	23.8	29.8	3.45	0.880	0.054	0.131

^1^ CP = corn processing (SFC vs. GC). ^2^ PD = protein degradability (HCP vs. LCP). ^3^ CP × PD = interaction between the factors. Values followed by different letters differ (*p* < 0.05) according to the F test. The use of bold indicates the variables that show a statistically significant level (*p*-value < 0.05).

**Table 3:** Particle selection index (% consumed/offered).

Particle Fraction	SFC-HCP	SFC-LCP	GC-HCP	GC-LCP	SEM	*p*-Value CP ^1^	*p*-Value PD ^2^	*p*-Value CP × PD ^3^
>19 mm (long particles)	96.1	97.2	87.4	90.9	1.5	**0.0009**	0.278	0.570
8–19 mm (intermediate particles)	100.5	99.5	100.8	100.5	0.2	0.096	0.112	0.393
1.18–8 mm (short particles)	100.8	100.6	100.8	100.5	0.2	0.988	0.384	0.908
<1.18 mm (very short particles)	100.6	101.2	101.6	100.7	0.4	0.615	0.834	0.120

^1^ CP = corn processing (SFC vs. GC). ^2^ PD = protein degradability (HCP vs. LCP). ^3^ CP × PD = interaction between the factors. The use of bold indicates the variables that show a statistically significant level (*p*-value < 0.05).

## Data Availability

The original contributions presented in this study are included in the article/Supplementary Materials. Further inquiries can be directed to the corresponding author.

## Supplementary Materials

The following supporting information can be downloaded at: https://www.mdpi.com/article/10.3390/ani16010107/s1, Figure S1: Effect of corn processing and crude protein degradability on total short-chain fatty acid (SCFA) concentration. Figure S2: Effect of corn processing and crude protein degradability on ruminal concentration of short-chain fatty acids and branched-chain fatty acids. Figure S3: Effect of corn processing and crude protein degradability on fecal and urinary pH.
