# Supplementary material for: Effect of Corn Processing and Protein Degradability on Ruminal Metabolism and Feeding Behavior of Dairy Cows
Source: Animals (Basel). 2025 Dec 30;16(1):107. doi: 10.3390/ani16010107 (PMC12784719; doi:10.3390/ani16010107)

Figure S1. Effect of corn processing and crude protein degradability on total short-chain fatty acids (SCFA) concentration

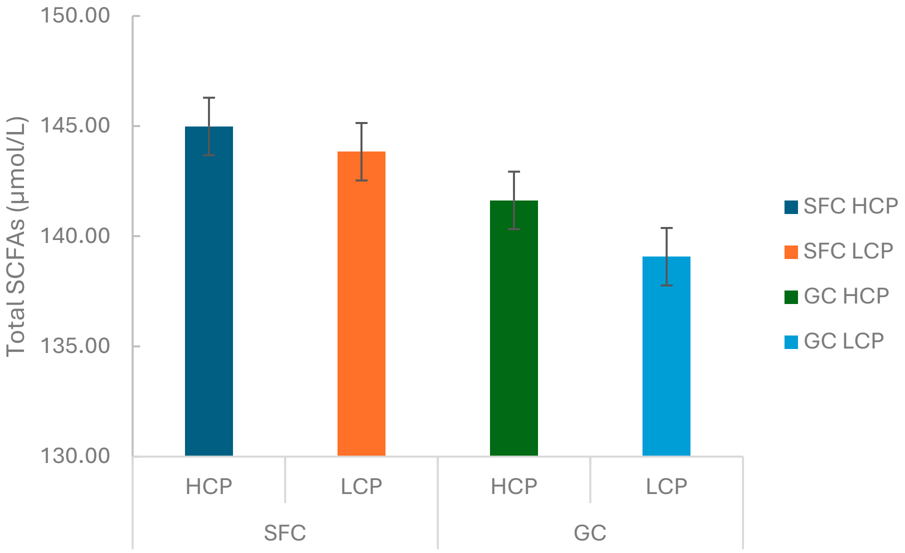

Figure S2. Effect of corn processing and crude protein degradability on ruminal concentration of short-chain fatty acids and branched-chain fatty acids

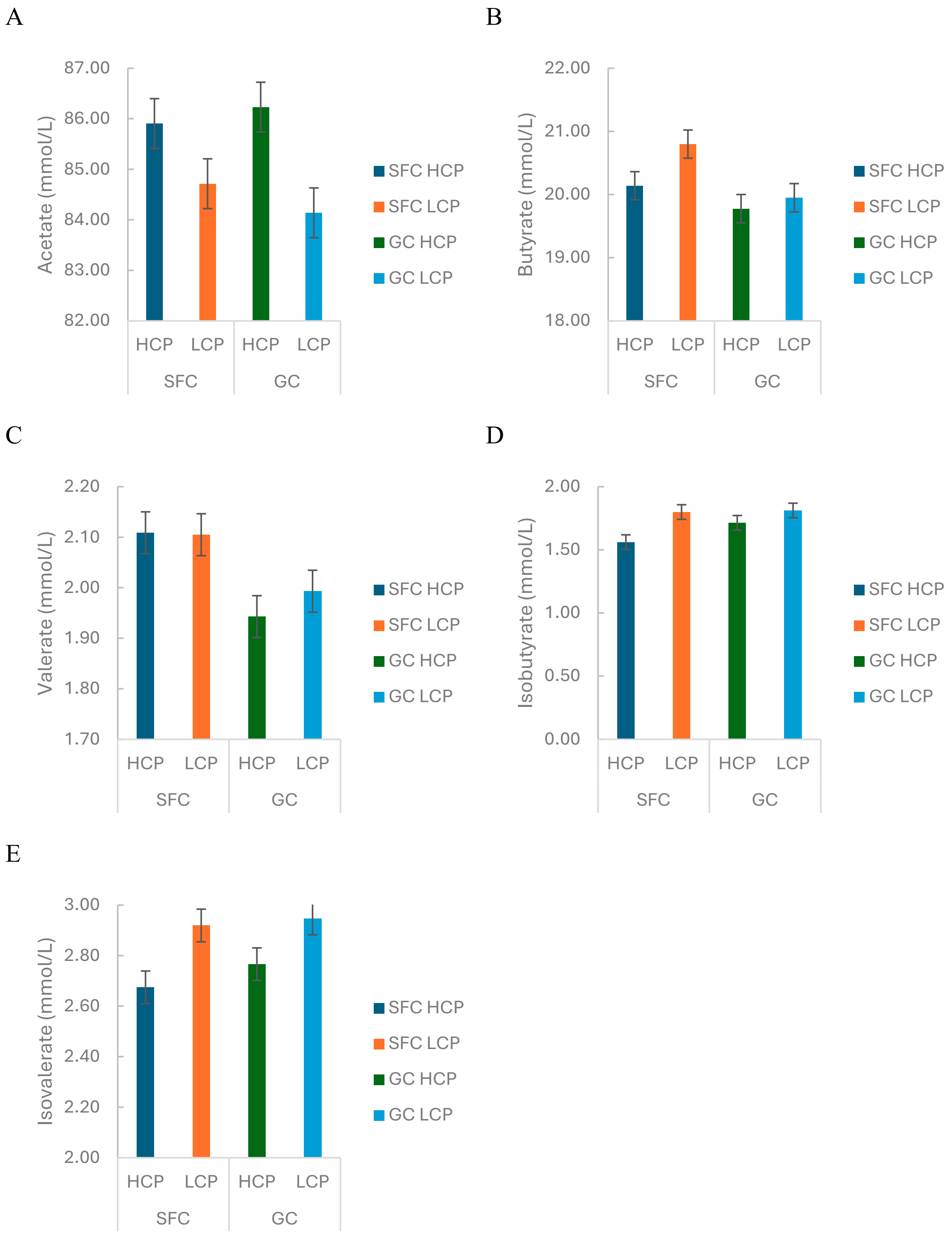

Figure S3. Effect of corn processing and crude protein degradability on fecal and urinary pH

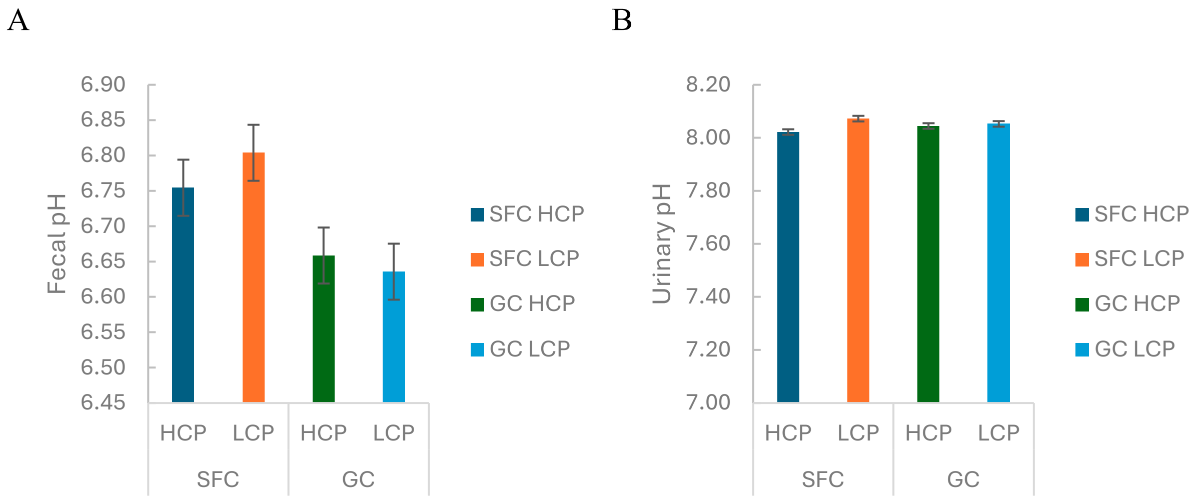

Supplement: Supplementary file 1 [file animals-16-00107-s001.zip › animals-3995279-supplementary.pdf]
